# Low-Power Laser Powder Bed Fusion Processing of Scalmalloy^®^

**DOI:** 10.3390/ma15093123

**Published:** 2022-04-26

**Authors:** Alessandra Martucci, Alberta Aversa, Diego Manfredi, Federica Bondioli, Sara Biamino, Daniele Ugues, Mariangela Lombardi, Paolo Fino

**Affiliations:** 1 Department of Applied Science and Technology, Politecnico di Torino, Corso Duca degli Abruzzi 24, 10129 Torino, Italy; alberta.aversa@polito.it (A.A.); diego.manfredi@polito.it (D.M.); federica.bondioli@polito.it (F.B.); sara.biamino@polito.it (S.B.); daniele.ugues@polito.it (D.U.); mariangela.lombardi@polito.it (M.L.); paolo.fino@polito.it (P.F.); 2 Consorzio Interuniversitario Nazionale per la Scienza e Tecnologia dei Materiali (INSTM), Via G. Giusti 9, 50121 Firenze, Italy; 3 Center for Sustainable Future Technologies IIT, Italian Institute of Technology, Via Livorno 60, 10144 Turin, Italy

**Keywords:** Al-based alloys, Scalmalloy, Al-Mg-Sc-Zr, Al_3_(Sc, Zr) phase, low power, parameter optimization, heat treatment, LPBF, additive manufacturing

## Abstract

Among recently developed high-strength and lightweight alloys, the high-performance Scalmalloy^®^ certainly stands out for laser powder bed fusion (LPBF) production. The primary goal of this study was to optimize the Scalmalloy^®^ LPBF process parameters by setting power values suitable for the use of lab-scale machines. Despite that these LPBF machines are commonly characterized by considerably lower maximum power values (around 100 W) compared to industrial-scale machines (up to 480 W), they are widely used when quick setup and short processing time are needed and a limited amount of powder is available. In order to obtain the optimal process parameters, the influence of volumetric energy density (VED) on the sample porosity, microstructure and mechanical properties was accurately studied. The obtained results reveal the stability of the microstructural and mechanical behaviour of the alloy for VEDs higher than 175 Jmm^−3^. In this way, an energy-and-time-saving choice at low VEDs can be taken for the LPBF production of Scalmalloy^®^. After identifying the low-power optimized process parameters, the effects of the heat treatment on the microstructural and mechanical properties were investigated. The results prove that low-VED heat-treated samples produced with an LPBF lab-scale machine can achieve outstanding mechanical performance compared with the results of energy-intensive industrial production.

## 1. Introduction

Additive manufacturing (AM) techniques are powerful tools that can provide significant potential for lightweight applications with a high degree of complexity in several industrial sectors. A fast-growing AM technology is laser powder bed fusion (LPBF). The LPBF process consists of the repeated generation of thin layers of metal powder and selective melting of the part cross-sections through a laser beam. Among the outstanding features of the LPBF technique, it is possible to mention near-to-shape production, short time-to-market and high mass saving. However, the achievable mass reduction depends not only on the process but also on the mechanical properties of the materials used [1].

The need to find high-strength and lightweight alloys resulted in a growing interest in aluminium alloys processable by the LPBF technique [2]. However, processing Al-based alloys by LPBF is a challenging task due to their high laser reflectivity, high thermal conductivity and high tendency to oxidation [3].

In the current state of the art of LPBF, AlSi10Mg, AlSi7Mg and AlSi12 surely emerge as the most used compositions. The LPBF process of these quasi-eutectic alloys is simplified by their thin solidification interval, which results in a lower risk of cracking. Moreover, according to Olakanmi et al. and Aversa et al., fairly good mechanical performances can already be obtained in the as-built state [4,5]. Notwithstanding this, Al-Si-based alloys produced with LPBF cannot satisfy the criteria for some specific applications due to limited strength, ductility and heat stability. The development of new high-performing aluminium alloys is, therefore, crucial [6].

To achieve outstanding properties, several attempts are currently being made using Sc- and Zr-modified Al-Mg alloys [5,7,8,9,10,11,12,13,14,15,16,17,18,19,20,21,22]. The effects of Sc on the properties of Al alloys have been investigated by several researchers. The strong interest in this alloying element is due to its ability to be a good inoculant for Al alloys creating very fine grains and the limited-solubility eutectic diagram it forms with aluminium [7]. The high-temperature Al-Sc solid solution, in fact, can decompose during cooling, resulting in finely dispersed and fully coherent Al_3_Sc intermetallic precipitates. Moreover, these Al_3_Sc precipitates are known to be very effective to prevent recrystallization even at high temperatures [8]. As reported by Davydov et al., no crystallographic modifications or loss of coherence with the matrix are recorded over a wide temperature range for the Al_3_Sc precipitates [9]. In order to further stabilise these precipitates, it is possible to add zirconium, which replaces the scandium atoms in Al_3_Sc resulting in Al_3_(Sc, Zr) having further-reduced coarsening kinetics [10]. In addition, the presence of Zr allows the reduction of the Sc concentration required to achieve the desired strengthening effect, resulting in relevant cost saving [9].

Within this framework, Scalmalloy^®^ has been developed and patented by APWORKS with the goal of obtaining a high-strength aluminium alloy with improved elongation, good corrosion resistance and weldability compared to the traditional Al alloys [11]. The use of Scalmalloy^®^ appears therefore to be promising for space components, possibly replacing titanium in high-strength and high-thermal-conductivity applications [12].

The peculiar microstructure of Scalmalloy^®^ has attracted widespread interest in the literature. In particular, different published works paid attention to the very fine bimodal grain size distribution that characterises this alloy in the as-built condition [8,13,14]. The high number of fully coherent Al_3_(Sc, Zr) precipitates on the melt pool boundary provides ideal nucleation sites that result in the simultaneous growth of fine and equiaxed Al grains [14]. On the contrary, a low number of precipitates in the centre of the melt pool results in coarser and more elongated grains in the direction of the thermal gradient [8]. Electron backscatter diffraction (EBSD) data demonstrated that, indeed, the microstructure is made of a network of interconnected melt pools containing fine and coarse grain regions. Detailed analyses of the grains revealed that in Scalmalloy LPBF parts, the coarser grains are 5 to 10 times smaller compared to the traditional Al alloys processed by LPBF [13].

Much research on LPBF Scalmalloy also focused on the post-processing heat treatment that leads to the highest mechanical properties [15,16,21,22]. Schmidtke et al., for example, reported the LPBF production of an Al-4.5Mg-0.66Sc-0.37Zr alloy (ScalmalloyRP^®^) and used an ageing treatment condition (325 °C for 4 h) that leads to a tensile strength of over 520 MPa [15]. Later, the effects of different heat treatments on the properties of LPBF Scalmalloy^®^ were also investigated by Spierings et al. who confirmed that a tensile strength exceeding 520 MPa can be obtained through ageing treatments performed at temperatures ranging from 325 to 350 °C for 4–10 h [16].

However, it has been widely demonstrated in the literature that the mechanical properties of LPBF parts are closely related to the process parameters used not only because of the level of densification achieved but also because of the microstructure generated due to different solidification conditions [17]. Considering the need to produce bulk samples without cracks and fusion defects, in the literature, there are some studies focused on optimizing the LPBF process parameters of Scalmalloy^®^. Nevertheless, the published works are focused exclusively on high-power optimisation, with power values ranging from 200 to 480 W [6,8,13,17,18]. These power values are not, however, suitable for the use of LPBF lab-scale machines.

Lab-scale machines are extremely useful in the LPBF research field since they are characterised by quick setup, short processing time and a limited amount of powder needed. The aim of this work was to enable the production of Scalmalloy^®^ also with lab-scale machines and thus to carry out a low-power optimisation. Boosting scanning productivity by lowering the energy density while maintaining mechanical characteristics was also an important focus of this research. Furthermore, an accurate study of the influence of heat treatment on microstructural evolution and mechanical properties was carried out.

## 2. Materials and Methods

### 2.1. Materials

A commercial gas-atomized Scalmalloy^®^ powder supplied by LPW (LPW Technology, Runcorn, UK) was selected for this study. The Scalmalloy^®^ composition measured by LPW (LPW Technology, Runcorn, UK) is reported in Table 1.

**Figure 1:**
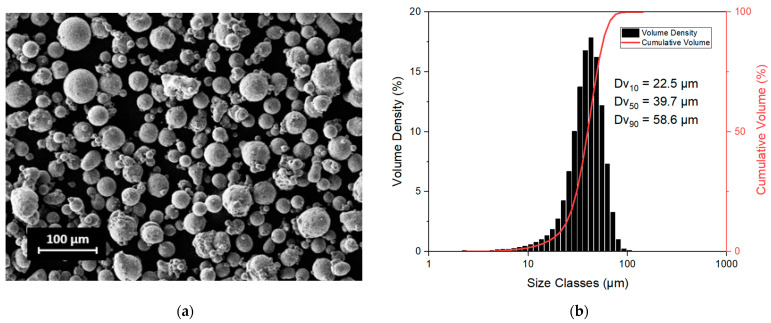
Powder morphology observed with SEM (**a**) and particle size distribution (**b**).

As it is possible to note in the scanning electron microscope (SEM) micrograph taken with a Tescan S9000G FIB (Tescan company, Brno, Czech Republic) (Figure 1a), particles present a spherical shape with some satellites. Moreover, the particle size distribution measured using a Malvern Mastersizer 3000 (Malvern Panalytical, Malvern, UK) is reported in Figure 1b together with the Dv_10_, Dv_50_ and Dv_90_ values.

As suggested by Concept Laser, before the LPBF process, the powder was sieved between 20 and 50 μm and then dried at 90 °C for 2 h to remove the residual moisture and improve the flowability. In fact, the correlation between the moisture on the powder surface and a higher hydrogen porosity after the LPBF process has been extensively verified in literature [23].

### 2.2. Production of LPBF Samples

The LPBF production was performed using a Concept Laser Mlab cusing R (General Electric, Boston, MA, USA). This is a lab-scale system with a 9 × 9 cm^2^ platform equipped with a fibre laser having 100 W maximum power, 1070 nm wavelength and a 50 μm spot size. Above all, the first investigation of the process parameters was carried out using 10 × 10 × 10 mm^3^ cubic samples produced onto an Al substrate. All samples were produced at 95 W (*P*), with a layer thickness of 15 μm (*L*) and a hatching distance of 105 μm (*hd*). The laser scan speed (*v*) was varied between 200 and 800 mms^−1^ resulting in a volumetric energy density (VED) range from 75 to 300 Jmm^−3^ applying Equation (1).

(1) $$VED=\frac{P}{l\times hd\times v},$$

A further job was performed using two VED conditions in order to carry out a more in-depth characterisation of the as-built and heat-treated states. The job included the production of cubic samples 10 × 10 × 10 mm^3^ for microstructural analysis and microhardness evaluation and bars 50 × 20 × 4 mm^3^ for Young’s modulus evaluation through an impulse excitation analysis. The bars were produced horizontally with the main dimensions parallel to the build platform. According to the procedure described by Schmidtke et al. and by Spierings et al., the heat treatment was performed at 325 °C for 4 h [15,16]. This heat treatment permits one to obtain precipitation of fine dispersed Al_3_(Sc, Zr) precipitates achieving the maximal material strength (R_m_ > 520 MPa and R_P0.2_ > 480 MPa).

### 2.3. Characterization

The as-built specimens were removed from the platform using an electrical discharge machine. All samples were cut in the middle along the building direction, ground and polished using standard metallographic procedures. Twenty optical micrographs (100× magnification) were taken unbiasedly across the XZ plane of the samples using a Leica DMI 5000 M optical microscope (Leica Microsystems, Wetzlar, Germany). All micrographs were processed using ImageJ software in order to obtain the percentage of pores and thus the relative density value of each sample. Phase identification was conducted using a PANalytical X-Pert diffractometer (Malvern Panalytical, Malvern, UK). The X-ray diffraction (XRD) analyses were recorded at 40 kV and 40 mA in a Bragg Brentano configuration, using a Cu Kα radiation. A step size of 0.013°, a time step of 25 s and a 2ϑ range between 30 and 100° were considered for the full diffractogram. After that, a detailed analysis of the first peak was conducted using 2ϑ = 37–39°, step size = 0.003° and time per step = 60 s. The microstructural analyses were performed using a Tescan S9000G FIB SEM (Tescan company, Brno, Czech Republic) equipped with an EBSD detector. EBSD orientation maps were recorded at 2.5× kX magnification and a step size of 110 nm. The SEM was operating at 20 kV and 10 nA, and the samples were tilted 70° and with a working distance of 5 mm.

The investigation of mechanical properties was performed by microhardness tests and by the calculation of Young’s modulus. The characterization of material hardness was conducted using a microhardness Vickers tester VMHT (Walter Uhl, Loherstraße, Germany) according to ASTM E384 standards. The microhardness analysis was carried out by applying a load of 0.1 kg and a dwell time of 15 s. Ten measurements for each sample were performed on the XZ plane. The dynamic Young’s modulus values were evaluated by the impulse excitation technique by an IMCE RFDA basic instrument (IMCE NV, Genk, Belgium) according to ASTM E1876 standards. By measuring resonance frequencies, this non-destructive test permits one to determine the Young’s modulus of a material of interest. Five measurements for each sample were carried out on the largest surface of the bars.

Finally, in order to analyse the phase transformation, a differential scanning calorimetry (DSC) analysis was then carried out on as-built and heat-treated samples using a Netzsch Polyma DSC 214 (NETZSCH group, Selb, Germany) differential scanning calorimeter in a pure Argon atmosphere. The heating and cooling rate was fixed at 20 °C/min, and the temperature was in the range of 20–540 °C.

## 3. Results

### 3.1. LPBF Process Parameter Window

**Figure 2:**
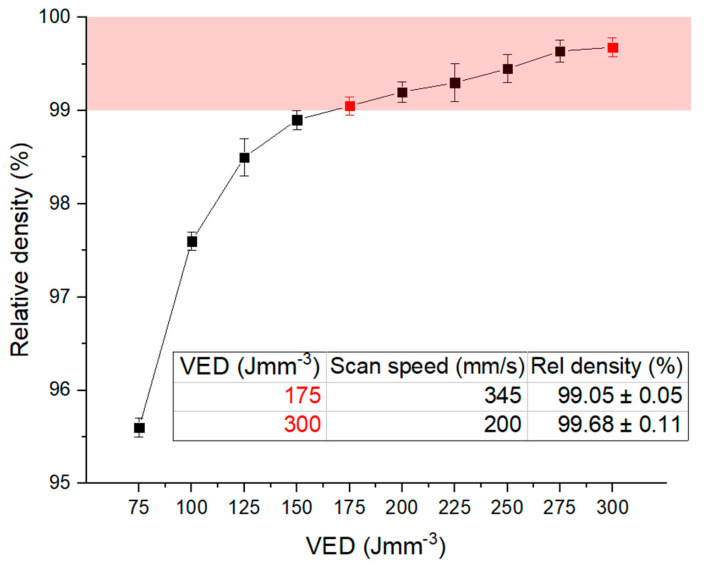
Relative density as a function of volumetric energy density. The optimal relative density zone (over 99%) is highlighted in red, and the two chosen VED conditions are marked and described in the table.

As highlighted with the red zone in Figure 2, the Scalmalloy^®^ samples built with VED values in the range of 175–300 Jmm^−3^ exhibit a remarkably high relative density (over 99%). For VED values above 250 Jmm^−3^, a plateau in relative density values was detected according to what was denoted by Spierings et al. [8,13]. Hence, a further increase in VED would result in poor productivity without a visible improvement in relative density. In order to compare the mechanical performances of materials produced with different energy densities, two different VED values providing a good relative density (marked in red in Figure 2) were selected: the lowest VED 175 Jmm^−3^ and the highest VED 300 Jmm^−3^. The selected VED values correspond to scan speeds of 345 and 200 mm/s, respectively.

### 3.2. Phase Identification

**Figure 3:**
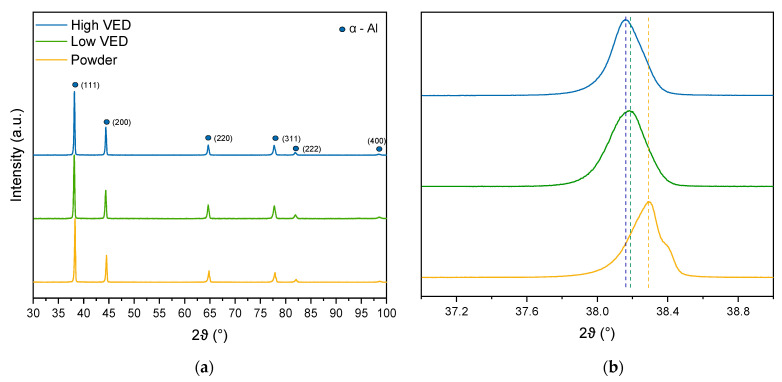
The complete XRD patterns of the raw powder, low- and high-VED as-built samples (**a**) and detailed analysis conducted on the (111) peak (**b**).

A preliminary XRD investigation was conducted on the raw powder and on high- and low-VED as-built samples. In Figure 3a, no significant differences in the patterns can be observed. In all cases, the Al_3_(Sc, Zr) phase was not detected due to its limited content in the matrix. In fact, the presence of a phase below 1 wt % in the matrix is not distinguishable with this kind of investigation. Observing the detailed analysis conducted on the α-Al (111) peak (Figure 3b), a shift to lower angles after LPBF processing can be noticed. Furthermore, no marked shift can be observed when the applied VED is changed.

### 3.3. As-Built Microstructure

**Figure 4:**
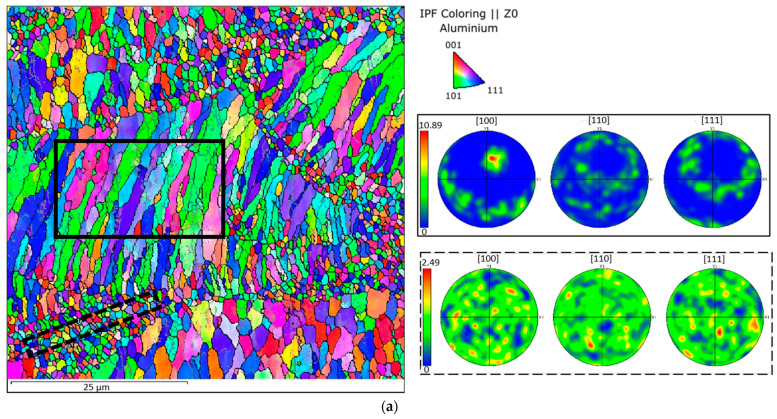

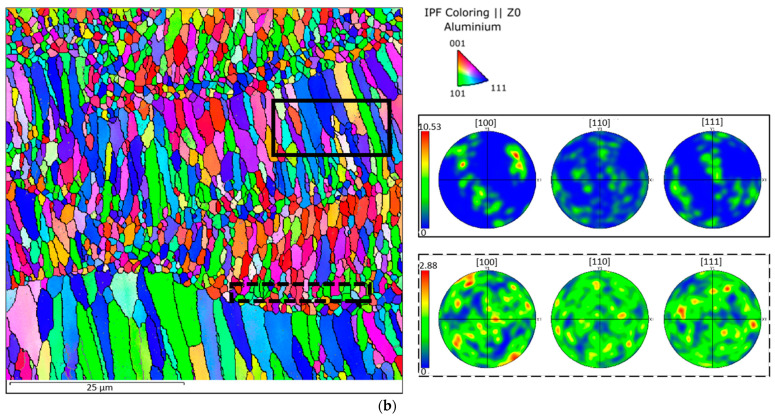
The EBSD orientation maps with pole figures related to CG and FG zones of low-VED (**a**) and high-VED (**b**) as-built samples.

The Scalmalloy^®^ as-built samples in both VED conditions are characterised by the typical LPBF microstructure made of a network of interconnected melt pools. In Figure 4, a layer-wise build structure can be observed with a very fine-grained (FG) microstructure, next to regions of coarser grains (CG). According to Spierings et al., this bimodal microstructure is due to the high number of coherent Al_3_(Sc, Zr) particles at the melt pool border that act as heterogeneous nucleation centres. On the contrary, in the centre of the melt pool, the lower number of particles leads to the solidification of coarser and more elongated grains that follow the thermal gradient.

In both VED conditions, the EBSD analyses highlighted a non-preferential orientation in the FG zone in contrast with the CG zone that shows a marked preferred orientation perpendicular to the melt pool boundary (Figure 4). Moreover, a more in-depth analysis of the grains revealed that the low- and high-VED microstructures contain a similar percentage of fine grains equal to 80 and 70%, respectively.

**Figure 5:**
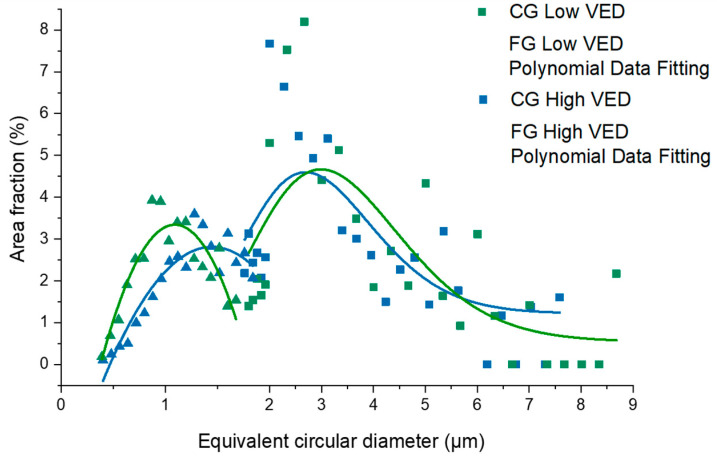
Grain size distribution for coarse (CG) and fine (FG) grain material for low- and high-VED as-built samples.

As reported in Figure 5, the equivalent circular diameters (ECDs) in the FG zones are very small in a range of 0.4 μm up to about 1.8 μm (both in low- and high-VED samples), although the coarser grains fluctuated between 2 and 9 μm. In the FG zone, the average grain size appears to be slightly smaller (1.1 μm) in the low-VED sample with respect to the high-VED sample (1.4 μm). In addition, comparing the ECD trends in the FG zones, it is possible to note the higher area fraction of the equiaxial grains in the low-VED sample. On the contrary, only extremely slight variations in grain distribution can be noted for the CG zone in the two VED conditions. In particular, only a slight shift is appreciable in the ECD trends in the CG zones, and a smaller fraction of grains with an ECD size greater than 6 µm can be detected for the low-VED sample.

### 3.4. As-Built Mechanical Properties

**Figure 6:**
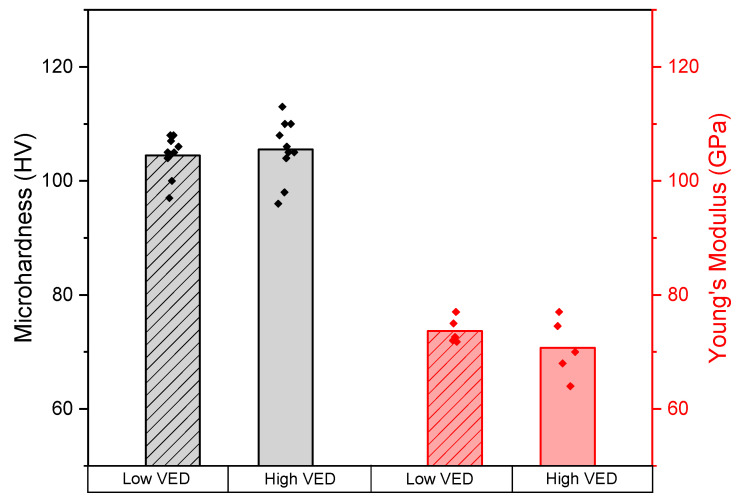
Results of microhardness and Young’s modulus tests for low- and high-VED as-built samples.

The mechanical properties were explored by performing microhardness tests and calculating Young’s moduli. As depicted in Figure 6, a mean microhardness value of about 105 HV was recorded on both samples. A limited fluctuation of 3 HV for the low-VED sample and 5 HV for the high-VED sample can be observed. Further analysis of the mechanical properties also reported in Figure 6 was carried out through Young’s modulus calculation. The Young’s modulus obtained for the low-VED sample is 74 GPa. With the increase in VED, a slight decrease in stiffness was recorded (70 GPa). The standard deviation becomes a relevant factor to consider. The low-VED sample has a smaller standard deviation and thus remains statistically more stable compared to the sample produced with higher VED.

### 3.5. Heat Treatment Effect

Considering the moderately finer microstructure and the slightly higher Young’s modulus, the effect of the heat treatment was studied only on the low-VED sample. The low-VED condition, in fact, allows the use of a 70% higher scan speed value, ensuring a considerably shorter processing time without a decrease in properties.

According to the data reported by Schmidtke et al. and Spierings et al., the heat treatment was carried out at 325 °C for 4 h [15,16]. Phase identification by XRD analysis conducted on the heat-treated sample did not show any difference compared to the pattern of the as-built condition (for this reason, the XRD pattern is not reported).

**Figure 7:**
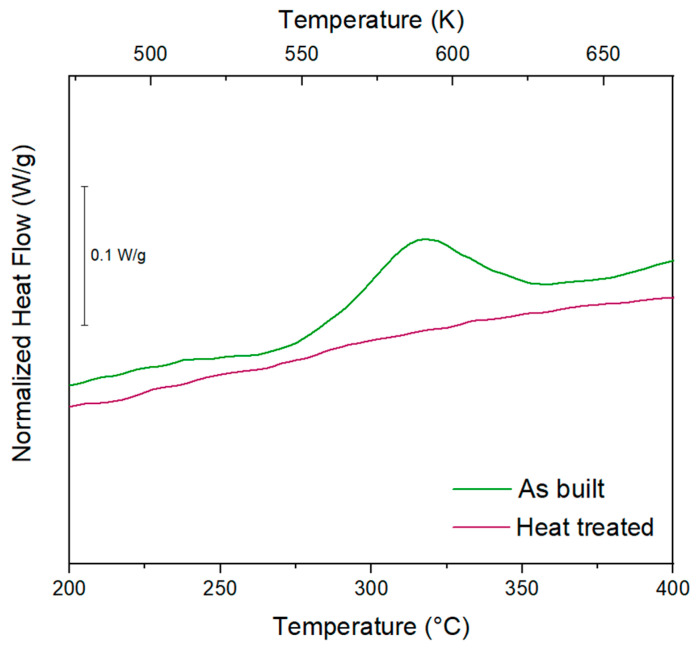
DSC signals of the as-built and heat-treated samples.

The material thermal behaviour and the heat treatment efficiency were investigated by performing DSC analysis on the as-built and heat-treated samples. Observing the DSC scans reported in Figure 7, one exothermic signal can be identified in the as-built sample. The exothermic nature of the peak indicates that some precipitation occurred between 268 and 355 °C. As evidenced by Vlach et al. [20], this signal is traceable to Al_3_(Sc, Zr) formation. After treating the sample at 325 °C for 4 h, this peak completely disappeared, suggesting that the Al_3_(Sc, Zr) precipitation completely occurred during the ageing heat treatment.

**Figure 8:**
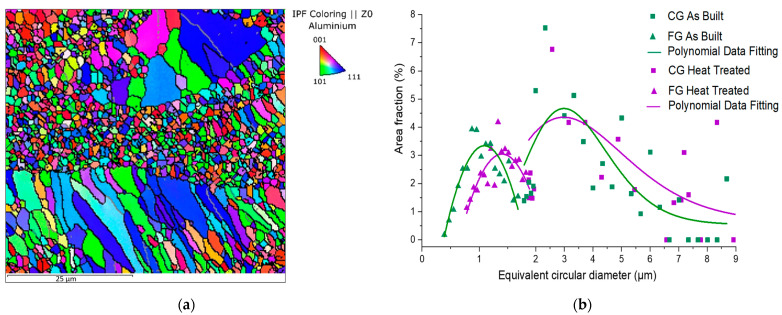
The EBSD orientation map of heat-treated sample (**a**) and grain size distribution before and after heat treatment (**b**).

In order to investigate the influence of ageing treatment on the microstructure, a further EBSD analysis was carried out on the heat-treated sample (Figure 8). From the comparison of the grain size distribution in the as-built (Figure 4) and heat-treated samples, it is possible to note that the heat treatment had a different effect on the FG and CG zones. As illustrated in Figure 8b, in the FG zone, the heat treatment appears not strictly correlated with the area fraction change. However, an increase of 40% in the mean ECD value can be observed for the heat-treated sample in comparison to the as-built one. The heat treatment effects are also evident in the CG zone where the trend of the graph indicates that the size of the columnar grains grew considerably. In particular, the area fraction of grains with an ECD greater than 4 µm is 20 and 30% for low and high VED, respectively.

**Figure 9:**
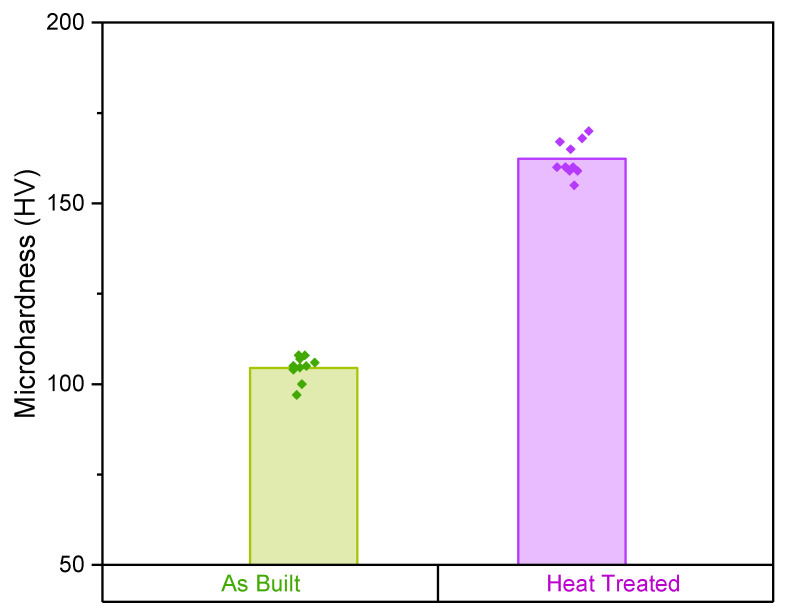
Results of microhardness Vickers analysis before and after heat treatment.

From a mechanical point of view, a mean microhardness value of 162 HV was measured for heat-treated samples, 50% higher than that of the as-built material (Figure 9). Moreover, as expected, no differences in Young’s modulus were observed after the heat treatment.

## 4. Discussion

With the aim of an accurate Scalmalloy^®^ low-power LPBF process optimisation, a wide spectrum of process conditions was analysed in terms of relative density. In particular, the VED was varied between 75 and 300 Jmm^−3^ because the majority of VED conditions explored in the literature are included in this range [6,8,13,14,15,16,17,18]. However, in this work, a power value of 95 W was set as compatible with an LPBF lab-scale machine use, and only the scan speed values were varied in order to achieve the desired energy range. In line with Springers et al.’s results, all samples realized with a VED greater than 175 Jmm^−3^ showed a very good level of densification (above 99%) [13]. From the samples that presented good densification, the highest and lowest VED conditions (i.e., 175 Jmm^−3^ and 300 Jmm^−3^, corresponding to scan speeds of 345 and 200 mm/s, respectively) were chosen for a comparison.

The microstructural and mechanical investigations conducted through XRD, EBSD, hardness and impulse excitation analyses revealed no marked differences between the two process conditions.

First of all, in the XRD results, a shift to low angles of the Al peaks detected in the powder was noticed after processing. According to Li et al., this shift can be correlated with the extremely high cooling rate during LPBF solidification [19]. The extremely high cooling rate during solidification has in fact led to a significant increase in the content of Mg/Sc/Zr atoms within the Al lattice, resulting in a higher plane distance [6]. According to Bragg’s law (Equation (2)), in fact, an increase in *d* values results in lower recorded angles.

(2) 2×d×sinϑ=nλ ,

where *n* is the diffraction order and *λ* is the wavelength.

In addition, the XRD patterns of the bulk samples built with low and high VED did not show differences in the angle of the Al peaks. This suggests that when using different VED values, there was no significant variation in cooling rate causing a change in the lattice distance or a noticeable difference in the precipitation phenomena.

In previously published works, accurate TEM observations revealed the formation of the nanometric Al_3_Sc phase in the as-built state [13,21]. However, peaks identifying this phase were not detected by XRD analysis, probably due to their presence below 1 wt% (sensitivity limit of the XRD test) or their nanometric size. Looking at the detailed diffractogram of the feedstock material, the double peak relative to the kα2 radiation can be observed. This can be attributed to larger powder crystallites that lead to narrower peaks, making kα2 detectable. As expressed in Scherrer’s law, in fact, the size of crystallites is inversely proportional to the broadening of the peak. The absence of this double signal after LPBF processing suggests that this AM process leads to smaller crystallites with respect to the powders.

Through the EBSD investigations, an in-depth study on grain orientation and grain size distribution was carried out. As extensively discussed in the literature, by introducing transition metals and rare earth elements to the aluminium alloy compositions, a peculiar bimodal microstructure can be achieved [5,24,25,26,27,28]. EBSD analyses highlighted, in fact, an FG zone at the melt pool border with equiaxial grains and a central CG zone with columnar grains. In Figure 4, the pole figures showed a preferential orientation in the [100] direction in agreement with Spierings et al. [8]. The [100] direction is parallel to the building direction and is consistent with the typical steep thermal gradient generated along this direction during the LPBF process [29]. The results of the EBSD analysis were also used to compare the fraction of equiaxial and columnar grains as a function of their ECD observed in the as-built samples produced with the two different process conditions. The analysis revealed a slightly finer microstructure for the sample produced at low VED. This phenomenon can be explained by the higher scan speeds involved (345 mm/s in the low-VED condition versus 200 mm/s in the high-VED one).

As far as the mechanical properties are concerned, no marked difference can be highlighted between the two VED conditions in terms of microhardness and Young’s modulus. In addition, the microhardness values recorded on LPBF Scalmalloy samples resulted in line with Spierings et al. and Li et al. and 30% higher than the cast-processed ones [13,19]. The improvement in mechanical properties compared to conventional technologies is the result of the finer microstructure that characterizes the additive processes [19]. As described by the Hall–Petch relationship, in fact, a fine microstructure results in improved tensile properties which are related by a factor of 1/3 to the hardness performance [30]. Furthermore, the obtained Young’s modulus values also proved to be perfectly in line with datasheet statements and with the values obtained by Spierings et al. using higher power values [11,13].

Considering the finer microstructure and the slightly higher Young’s modulus, it was decided to proceed with the post heat treatment only for the low-VED sample. These LPBF conditions, in fact, guarantee a 70% increase in building rate (Equation (3)) with respect to the high-VED parameters.

(3) Build rate=l×hd×v ,

The DSC comparison between the as-built and the heat-treated conditions permitted us to verify the heat treatment effectiveness. From the DSC analysis performed on the as-built sample, an exothermic peak centred at 312 °C and related to the Al_3_(Sc, Zr) formation can be identified. In addition, the completed Al_3_(Sc, Zr) precipitation reactions were confirmed by the disappearance of the exothermic peak after the heat treatment. Nevertheless, XRD analysis did not reveal the peaks of Al_3_(Sc, Zr) precipitates after the heat treatment due to their nanometric dimensions. After an accurate TEM investigation, Spierings et al. in agreement with Booth-Morrison et al. found an increased numerical density of Al_3_(Sc, Zr) in heat-treated samples but with a mean size of 3.2 nm [22,31]. As previously mentioned, the size affects the shape of the peak in the XRD pattern, and nanometric dimensions lead to very large peaks that are not distinguishable from the background noise of the measurement.

The comparison of the EBSD results (Figure 8) indicates a 10% increase in the area fraction of grains with a size greater than 4 µm after the heat treatment. An increase in grain size can be noted also for equiaxial grains, going from an ECD of 1.1 to 1.4 µm after heat treatment. However, it is reasonable to suppose that the grain growth was limited by the inoculating effect of the Al_3_(Sc, Zr) precipitates.

The main evidence of the completed Al_3_(Sc, Zr) precipitation reactions observed through the DSC analysis can be observed with the mechanical behaviour. It is well-known that adding pinning points that inhibit the motion of dislocations can strengthen the material by requiring higher applied stress to overcome the pinning stress and continue the movement of the dislocations [32,33]. A remarkable increase in hardness of more than 50% was obtained after the heat treatment resulting in values comparable with the declared performance of the datasheet. This increase can be correlated with the strengthening effect of the Al_3_(Sc, Zr) precipitates. In addition, the hardness value achieved in this study after heat treatment is higher than that observed by Li et al. using more than doubled power to produce the samples [21].

All these considerations indicate that the low-power LPBF process can produce bulk Scalmalloy parts having the same properties as high-power LPBF ones after heat treatment.

## 5. Conclusions

Different works on the Scalmalloy^®^ LPBF process parameter optimization have been published in recent years. Nevertheless, the power values used in the literature (from 200 to 480 W) are too high to be suitable for LPBF lab-scale machines. This category of LPBF machines is usually used when quick setup and short processing time are needed and when a limited amount of powder is available.

In this study, a process parameter optimisation was carried out with the aim of extending the Scalmalloy^®^ processing also to lab-scale machines. After setting the used power value to 95 W, the densification level was investigated varying the VED values in a range of 75 to 300 Jmm^−3^. In order to study the effect of the energy density on microstructural features and mechanical properties, several analyses were performed on samples produced with 175 and 300 Jmm^−3^, corresponding to scan speeds of 345 and 200 mm/s, respectively. When the low-VED condition was fixed, a further study of the effects of the heat treatment on microstructural and mechanical properties evolution was conducted.

The following conclusions can be drawn:

- An exponential increase in densification level from 95.6 to 99.7% was observed by increasing the volumetric energy density. Above 175 Jmm^−^^3^, however, there was a relative density stabilisation with values above 99%.
- EBSD analyses revealed a similar preferential growing direction in the CG zone for both VED conditions, showing an orientation parallel to the build direction. However, a general finer microstructure was observed for the low-VED sample.
- The mechanical investigations led to an identical mean microhardness value of 105 HV for each processing condition and a slightly higher Young’s modulus value for the low-VED sample (74 GPa).
- The comparison of DSC signals between the as-built and the heat-treated conditions permitted us to verify the effectiveness of the heat treatment conducted at 325 °C for 4 h. An exothermic peak centred at 312 °C and related to the Al_3_(Sc, Zr) formation was detected only for the as-built sample. Therefore, the disappearance of the latter for the heat-treated sample suggested the accomplishment of precipitation reactions.
- Despite the EBSD analysis, a fairly marked grain size change was highlighted after the heat treatment; the growth was controlled by the inoculating action of precipitates. In addition, an impressive improvement in mechanical properties was noticed after the heat treatment with an increase in microhardness of more than 50%.

The above results suggest that a low-power LPBF production can be easily achieved for Scalmalloy^®^, still guaranteeing a short processing time and high mechanical performance. Low-VED parameters allow a 72% increase in build rate, greatly accelerating the build process. This time gain has to be added to the fast setup of a lab-scale machine. The result is a remarkably time-saving and thus cost-effective optimisation. Furthermore, the low-VED heat-treated sample revealed a remarkable increase in microhardness resulting in a value comparable with the datasheet declared performances and literature results. The results demonstrate the possibility of using lab-scale machines to produce this promising alloy marking an important achievement in the new material development and in innovative design studies. In fact, the quick setup and limited powder volumes required by these machines provide endless possibilities for the R&D sector. Nevertheless, the small build volume does not allow mass production or large components.

## Figures and Tables

**Table 1:** Scalmalloy^®^ chemical composition.

Element (wt%)	Mg	Sc	Mn	Zr	Fe	Si	Other Elements
Scalmalloy^®^	4.77	0.78	0.51	0.27	0.12	0.06	<0.3
